# Makers or Labellers

**Published:** 1888-03-24

**Authors:** 


### The Hospital

A Weekly Institutional Journal of

**Science, Medicine, and Philanthropy.**

*For Hospitals, Asylums, Medical Practitioners, Students, Nurses, Families, Parishes, Congregations, and the Charitable Public.*

Vol. III. No. 78.] [March 24, 1888.

### Makers or Labellers.

For some time past there has been an agitation in the direction of creating a teaching university for London. At present the London University consists of a Board of Examiners, upon which Government has conferred the power of granting academical degrees. We are fallen upon times of competition, and competitors necessitate those who are to declare how many and which of the competing multitude are to receive prizes. Hence the existence of bodies of men whose only business it is to ask questions, receive answers, and mark values.

It cannot be said that the Examining Board which goes by the name of the "London University" has done nothing for education. It has at least inspected with care many of the finished products of various educational workshops, and has affixed more or less appropriate labels on them. This is a work of some value—indeed, of a good deal of value; but is it all which London is capable of doing, and all which London ought to do? It was a work which when first undertaken there was much need of, because there were then in various parts of the country many manufactories of scholars which could not get their finished products authoritatively labelled at all, and so many such products of high value had to pass through the world without hall-mark of any kind, to their frequent disadvantage. This is no longer the case. Any educational workshop, however humble, which thinks it can make scholars, may prove at once the nature of its capabilities by sending up its work to Burlington House to be adjudicated upon. For this advantage many very modest workers have reason to be grateful.

But now a question arises, which is constantly occurring to the practical mind: Whether is the greater function,—*The making of scholars* or *the labelling of them* when made? Some people will think the question not so easy to answer as it would appear. But perhaps we may be helped to answer it by asking ourselves one or two others. Could the world better spare its scholars or dispense with their labels? Really, so far as the world is concerned, it would not much matter whether there were any academical degrees at all; but it would matter very greatly if there were no scholars. Supposing it were suggested to do away with the title of farmer, gardener, or the like, would any real difference be made at the dinner table? But if anyone were to shoot all those who actually produce our bread and beef, our cauliflowers and potatoes, there would be a social eruption of a character decidedly Vesuvian. We may conclude then that the scholars are the real indispensable current coin, and that the hall-mark is a secondary consideration.

What is a scholar? What is London doing to make scholars? Is it a place eminently fitted for the making of scholars? Is it better fitted on the whole than any other place? These are questions which go to the roots of certain matters. Take one of them—What is a scholar? He is of two kinds: he is a creature who deals with words; or he is a creature who deals with things. Many people will object to the former of these definitions; but let it pass—it is sufficiently true for our present purpose. For the making of scholars whose principal business and pleasure lie with words London is not perhaps so suitable and efficient a manufactory as some other places—Oxford and Cambridge for example. But for the making of scholars whose pleasure and business are with things there is no place in the world more suitable than London. Is there any other place so suitable?

To begin with, there are more things of all kinds in London than can be found within the same area in any other place on earth; and, moreover, there are more persons in London who know what is to be known about the things, and who know it well, than can be found anywhere else. But surely these are the very primest essentials of scholar-making—a workshop full of things the knowledge of which constitutes scholarship, and a host of men on all sides who are brimming over with knowledge of the things. What more is wanted? The things are here; the teachers are here; the pupils are here. Where is the defect? There is no order in the workshop—no gradation. There is, in fact, no single highest class workshop. The places of instruction are legion. In some of them there may be found one teacher of first-rate eminence and capacity, but he will be associated with a dozen others of no special merit. The whole system is therefore like a huge beast who has four unequal legs. Progress is made, but it is slow and unwieldy to the last degree.

The worst fault of all to be found with the education of London is that many of its best and most capable teachers are not teaching at all, but are degraded to the function of "labelling" the work of other men. How is it that they can be content with such poor and comparatively worthless drudgery? Can any true teacher feel in his own mind that examining is for a moment to be compared with teaching? Is it possible to imagine Tennyson, or Carlyle, or Huxley, or even John Tyndall spending all his days as an examiner?

In this vast gathering of human beings and incomparable centre of human interests called London, there ought to be concentrated at a given point all the ablest teachers of every subject of human knowledge; then to that point all the ablest pupils from all other schools and colleges would converge as naturally as beams of light concentre themselves upon the eye; and that unique assembly of teachers and taught would appropriately be called the "London University." It seems to us absurd to call an examining board "an university"; it is still more absurd and injurious to squander and waste our teachers and material as we do for want of a comprehensive and systematic intelligence. From focussing, as we suggest, all the highest teaching capacity and enthusiasm at a given centre, and so drawing all the highest learning capacity to the same centre, there would result—what has never yet been seen in its highest perfection in all the world—a gathering of minds and a labour and conflict of intellectual energy worthy of bearing the noble designation of "University."

The scheme suggested will repay consideration. The practical fruits which would spring from its realisation would be of priceless worth. There are now numbers of teachers in London whose services are lost to the world because there is no open arena where their powers may be displayed. The few colleges and the many hospitals which exist are closed preserves where none may enter except by certain gateways to which men of the highest capacity will not always stoop. Let us have an open market of teaching as we have an open market of commerce, and very soon the intellectual wares of the country will be increased in value a hundredfold. If the teaching be thoroughly done the labelling may take care of itself.

